# The Impact of a Planned Change to Nurse Staffing Levels in Emergency Departments: A Pre‐Test, Post‐Test Design

**DOI:** 10.1111/jan.16845

**Published:** 2025-02-24

**Authors:** Vera J. C. Mc Carthy, Noeleen Brady, Ashling Murphy, Aileen Murphy, Jane Ball, Robert Crouch, Christine Duffield, Peter Griffiths, Anne Scott, Jonathan Drennan

**Affiliations:** ^1^ Catherine McAuley School of Nursing and Midwifery University College Cork Cork Ireland; ^2^ Department of Economics, Cork University Business School University College Cork Cork Ireland; ^3^ School of Health Sciences University of Southampton Southampton UK; ^4^ University Hospital Southampton Southampton UK; ^5^ Emeritus Professor Edith Cowan University Joondalup Western Australia Australia; ^6^ University of Technology Sydney Sydney New South Wales Australia; ^7^ University of Galway Galway Ireland; ^8^ School of Nursing, Midwifery and Health Systems University College Dublin Belfield Ireland

**Keywords:** burnout, emergency department, intention to stay, job satisfaction, nurse staffing, planned change, pre‐post test, work environment

## Abstract

**Aim:**

To examine burnout levels, nurse perceptions of the work environment, job satisfaction, intention to stay and quality of care for nurses working in emergency departments before and following a planned change to nurse staffing levels.

**Design:**

A pre‐post observational design.

**Methods:**

A systematic approach (Nursing Hours per Patient Presentation) was introduced to determine nurse staffing levels based on patient presentations resulting in adjustments to nurse staffing. Data on burnout, the work environment, intention to stay, job satisfaction and quality of care were collected from three emergency departments prior to and following the adjustments to nurse staffing.

**Results:**

An adjustment to nurse staffing levels was made to all three emergency departments. Mean emotional exhaustion scores were significantly lower, and quality of work environment scores and levels of job satisfaction were significantly higher for nurses following staffing adjustments. There was an increase to the proportion of nurses who perceived an improvement in quality of care delivered. In general, the results indicated improvements in outcomes following adjustments to nurse staffing levels.

**Conclusion:**

A more holistic organisational approach is required to address staffing in emergency departments. Initiatives that involve frontline nurses in resource planning facilitating a bottom‐up approach to allow for improved work environments would be beneficial.

**Impact:**

This study addressed a planned change to nurse staffing levels in emergency departments and staff outcomes pre and post changes to staffing levels.

This study highlighted that staffing an emergency department, based on nursing hours per patient presentation, was associated with improvements in staff outcomes.

The research will impact on nurses working in emergency departments as outcomes from this research were used to develop a Framework for Safe Nurse Staffing and Skill Mix in Emergency Care Settings.

**Reporting Method:**

STROBE and SQUIRE checklist.

**Patient or Public Contribution:**

No Patient or Public Contribution.

**Summary**

- What does this paper contribute to the wider global clinical community?
  ○ Staffing emergency departments based on patient acuity has a number of positive outcomes for nursing staff. One of these outcomes is the facilitation of the recruitment and retention of nursing staff to emergency departments.
  ○ Details on how to staff an emergency department based on patient acuity have been discussed in this paper with appropriate details relating to the Framework for Safe Nurse Staffing and Skill Mix in Adult Emergency Care Settings in Ireland.
  ○ An uplift to staffing did result in positive staff outcomes, however, there is a need for a wider organisational initiative to address staff burnout, ensure retention of nurses working in emergency departments and to reduce the loss of valuable expertise in these settings.

## 1 Introduction

The experience of negative work characteristics and lower staffing levels for nurses has been associated with burnout and this in turn leads to nurses leaving or intending to leave their post (Dall'Ora et al. 2020). Researchers have found that poor staffing levels, were associated with perceptions of poor leadership and inadequate nurse‐doctor relationships (Dall'Ora et al. 2020). These relationships have also been identified amongst nursing staff working in emergency departments (EDs) where, despite reports of relatively good levels of job satisfaction and engagement with work, emergency nurses have high levels of burnout (de Wijn et al. 2022). de Wijn et al. (2022) also reported that burnout domains such as, high emotional exhaustion and depersonalisation, were due mainly to the unpredictable nature of the emergency nurse's work. There is a suggestion that a bi‐directional effect is evident with poor staffing leading to emotional exhaustion. This in turn leads to high turnover, resulting in more staff shortages and higher demands on the remaining emergency nurses (Caulfield et al. 2023).

It is of note that the majority of the literature that measures the association between nurse staffing does so in relation to patient outcomes and originates from acute medical and surgical settings. There is a dearth of high‐quality literature focused on ED nurse staffing and staff outcomes (Drennan et al. 2024; Butler et al. 2019). What is known is that EDs are stressful environments for staff (Gómez‐Urquiza et al. 2017) that can result in nurses experiencing burnout. Burnout is a condition theorised as ensuing from unmanaged chronic workplace stress (World Health Organization 2019) which can impact on the quality of care delivered. Low or inadequate staffing levels, poor work environments, inadequate leadership and poor collegial relations all contribute to burnout, poor job satisfaction and intention to leave (Dall'Ora et al. 2020). There are a number of approaches that can be used to reduce burnout with safe staffing levels highlighted as one of the main processes that should be used (Norful et al. 2023; Aiken et al. 2024). Creating a positive work environment through staffing based on patient acuity, with senior staff in a supervisory role can reduce factors that lead to chronic workplace stress and thereby reduce burnout and enhance staff retention (Dall'Ora et al. 2020).

The experience of the nurse working in ED is determined somewhat by the needs of the presenting patients (Caufield et al. 2023). To improve ED nurse retention, organisations need to develop strategies, re‐organise structures and respond to the health needs of staff to improve job satisfaction. This in turn, would support the delivery of high‐quality care to the patients attending ED (Drennan et al. 2024; Department of Health 2022).

The purpose of this paper is to determine if there was a change in the burnout levels, perceived work environment, job satisfaction, intention to stay and quality of care delivered for nurses working in ED following a planned change to nurse staffing levels. These changes were based on the introduction of a systematic approach to determine the nurse staffing complement, centred on patient acuity. We measured burnout, perceived work environment, job satisfaction, intention to stay and quality of care pre and post a planned change to nurse staffing to determine if staffing adjustments impacted these outcomes for nurses in three adult ED settings in Ireland.

## 2 Background

The determination of safe and appropriate nurse staffing levels and skill mix in the ED has traditionally been based on historical need and professional judgement rather than being informed by a systematic structured approach (Youd 2015). In Ireland, as in other developed countries, there is increasing demand for emergency care with EDs being the first point of contact for most people seeking healthcare in the acute hospital setting. There has been an exponential growth in the number of presentations to EDs internationally, which is largely driven by an ageing population (Morley et al. 2018). Increased patient presentations which are compounded by restricted access to hospital beds, is resulting in overcrowding. This can have a negative impact on the working environment and the wellbeing of nurses in ED (McDermid et al. 2020).

A meta‐analysis published in 2017 reported that approximately 30% of emergency nurses showed evidence of burnout (Gómez‐Urquiza et al. 2017). A number of factors have been identified as being associated with burnout and intention to leave including a poor work environment (Dall'Ora et al. 2020; McDermid et al. 2020). In addition, the COVID‐19 pandemic added to the work stress and burnout of nurses possibly leading to the loss of highly experienced staff from the profession (Buchan and Catton 2023).

The issues that relate to a poor working environment in ED include, crowding, workloads associated with a high patient volume and staffing levels that do not match patient need (McDermid et al. 2020; Norful et al. 2023). The work environment of the ED nurse needs to be a place that facilitates the delivery of high quality care and augments the nurse's ability to perform their role skilfully (Lake 2002). It has been reported that organisations with a good work environment have lower levels of burnout in nurses and there are recommendations for system‐level initiatives to improve the environment in which nurses practice (Buchan and Catton 2023). Despite these calls for system‐level change, it has been acknowledged that, due to the multiplicity of demands on ED nurses, the work environment is showing little sign of improving (McDermid et al. 2020). One of the main factors in determining a quality working environment for nursing staff in ED, and an area that can be modified, is ensuring staffing resources reflect patient acuity. Although it has been acknowledged that to determine safe staffing levels an accurate, system‐level record of patient acuity is required (Al‐Dweik and Ahmad 2020); this has been a challenge in EDs for a multitude of factors including the variability in patient flow and patient demand.

There are a number of approaches that can be used to determine safe nurse staffing levels in ED. For example, Wise et al. (2015) recommended the use of nurse to patient ratios in EDs to address understaffing with departmental discretion used in the allocation of the nurses based on patient need. However, these authors caution that nurse patient ratios cannot establish the ideal staffing levels for each clinical environment. Therefore, there is a need to determine ED nurse staffing levels based on patient need and to measure the extent, these staffing levels can reduce nurse burnout, improve work environments and increase care quality and job satisfaction (Department of Health 2022).

Based on the recognition that there is a need to improve the working environment for nursing staff in EDs, the Department of Health published *The Framework for Safe Nurse Staffing and Skill Mix in Adult Emergency Care Settings in Ireland* (Department of Health 2022). This Framework recommended a systematic approach to determine safe nurse staffing levels according to the hours of nursing care patients required. This led to the introduction of determining staffing levels in ED based on Nursing Hours required per Patient Presentation (NHpPP); these hours were based on weightings according to the patients' Manchester Triage System score on presentation to the ED (Mackway‐Jones et al. 2006). Prior to the introduction of NHpPP, EDs in Ireland were generally staffed using professional judgement and historical decision making. The impact of the introduction of this systematic approach to determine nurse staffing levels in ED will be examined in this paper. There were also adjustments to healthcare assistants (HCAs) staffing levels in the EDs; however, for the purpose of this paper, only registered nurses (RNs) responses are considered.

## 3 The Study

The purpose of this study was to determine if there was a change in the burnout levels, perceived work environment, job satisfaction, intention to stay and quality of care delivered for nurses working in three adult ED settings after a planned change to nurse staffing levels. These changes were based on the introduction of a systematic approach to determine the staffing complement, centred on patient need (i.e., NHpPP).

### 3.1 Aim

This study aimed to examine the impact of a planned change to nurse staffing levels based on patient need on the levels of burnout, perceived work environment, job satisfaction, intention to stay and quality of care delivered, for nurses, working in the ED.

The study objective was to determine burnout levels, perceptions of the work environment, job satisfaction, intention to stay and quality of care as reported by nurses working in ED pre and post a planned change to nurse staffing levels.

### 3.2 Intervention

The Framework for Safe Nurse Staffing and Skill Mix in Adult Emergency Care Settings in Ireland, published by the Department of Health (2022), recommended the introduction of a systematic approach to determine nurse staffing levels (NHpPP), a skill mix of 85% RNs to 15% HCAs and that the senior nurse in charge of the ED on each shift worked in an entirely supervisory role (i.e., they do not take on a direct patient caseload). The calculation of NHpPP was based on the number of patient presentations to the ED and their triage category; this allowed for the number of full‐time equivalent (FTE) nursing staff required for the EDs to be identified. The Manchester Triage System (Mackway‐Jones et al. 2006) was used to determine the level of urgency of care for patients presenting to ED; triage was performed by RNs. The number of triage nurses in the ED was based on the number of patients presenting to the ED annually with the recommendation that an ED with over 40,000 patient presentations annually should have a minimum of two RNs allocated to triage (Department of Health 2022).

To calculate the NHpPP, the annual number of patients presenting to the ED were separated by triage category. There were five triage categories: immediate; very urgent; urgent; standard and non‐urgent, weighted as per the mean number of hours of care per category which were: 6.13 immediate; 3.83 very urgent; 2.33 urgent; 1.42 standard and 0.58 non‐urgent. For example, a patient in the immediate triage category required 6.13 h of ED care; if there were 500 patients per year presenting to the ED in this category, the total number of nursing hours required for those categorised as immediate would be 3065 (6.13 × 500). The total number of nursing care hours required by triage category is then calculated and additional staff for triage are included based on the number of patients presenting to the ED annually. The gross nursing hours (RNs and HCAs) are then calculated (FTE) based on the standard weekly working hours (working week of 39 h for 52 weeks). A staff replacement rate to cover study leave/holidays/absences of 20% is added in addition to the percentage maternity leave for that ED. This provided the recommended FTE which was then divided in a ratio of 85%:15% RNs to HCAs FTEs (Department of Health 2022). These calculations identified that, in total, the three EDs where the model was tested were found to require an upward adjustment in RN staffing with a total of 27.91 FTE RNs across the sites (ED 1 addition of 2.11 FTE, ED 2 addition of 7.1 FTE and ED 3 addition of 18.7 FTE) (Department of Health 2022).

## 4 Methods

### 4.1 Design

The study used a pre‐post observational design.

### 4.2 Sample

All RNs (staff nurses and clinical nurse managers who had direct patient contact) working in three EDs were included in the study. Those eligible to take part in the study included RNs all of whom were directly employed by their respective organisation; agency nursing staff were excluded. There was no restriction in relation to time working as a qualified nurse. In total, 124 RNs provided data at Time 1 (before the introduction of the Framework) giving a response rate of 56.8% and 129 RNs provided data at Time 2 (after the introduction of the Framework) with a response rate of 58.6%.

### 4.3 Data Collection

A pre‐post study design was put in place in three EDs in the Republic of Ireland from November 2018–July 2020. Data were collected from staff nurses in ED at Time 1 (November/December 2018) before the adjustments to staffing which occurred throughout 2019 in the three EDs, and Time 2 after the adjustments (February/April 2020). Staff changes did not occur simultaneously at all three EDs owing to recruitment issues but typically the adjustments were made between October and December of 2019. All adjustments were in place for at least 2 months by February 2020 when Time 2 data collection began.

### 4.4 Procedure

Respondents were required to complete a questionnaire delivered to each eligible member of staff in the three EDs. An approach designed by Dillman (1978) was used to maximise response rates including multiple contacts with staff and personalised and targeted reminders. In addition, the research team visited the research sites and met with the nursing staff to explain the purpose of the study. Staff were provided with the opportunity to place the completed questionnaires in a secure container in the ED or return them by post directly to the research team. Staff questionnaires were coded to allow for linking of data from the two time‐points. As there were additional staff in the EDs at Time 2, these responses were also included in the final analysis. The data set was pseudo‐anonymised on data entry and then fully anonymised on completion of the data entry. A number of outcomes, including staff experience of burnout, RNs' perceptions of the working environment, job satisfaction, intention to stay and quality of care were measured both prior to and following the introduction of the recommendations in the Framework.

### 4.5 Measures

Burnout was measured prior to and following the introduction of the Framework (adjustment of staffing levels, skill mix and supervisory role of the shift leader) using the Maslach Burnout Inventory (MBI)—Human Services Survey Medical Personnel (MBI‐HSS MP) (Maslach and Jackson 1996). This 22‐item instrument measures burnout across three domains: emotional exhaustion (9 items); depersonalisation (5 items); lack of personal accomplishment (8 items). The emotional exhaustion subscale measures feelings of emotional overextension from work. Impersonal response to receivers of care is measured by the depersonalisation subscale and feelings of achievement and capability in your work are assessed in the personal accomplishment subscale. All 22‐items are rated on a 7‐point scale with 0 = never to 6 = every day. The sum of the nine items for emotional exhaustion gave a theoretical range of 0–54; for the five depersonalisation items a theoretical range of 0–30 and 0–48 for the eight items measuring personal accomplishment. High scores in emotional exhaustion and depersonalisation and low scores in personal accomplishment suggest negative feelings conceptualised as burnout. The MBI has been reported to have Cronbach's alpha scores above 0.70 for each of the three domains (Maslach and Jackson 1996). In this study, Cronbach's alpha for the MBI‐HSS MP were, emotional exhaustion—Time 1 α = 0.71/Time 2 α = 0.89; depersonalisation—Time 1 *α* = 0.75/Time 2 *α* = 0.73; lack of personal accomplishment—Time 1 *α* = 0.66/Time 2 *α* = 0.73.

The work environment of ED nurses was assessed using the Practice Environment Scale of the Nursing Work Index—(PES–NWI‐R) (Aiken and Patrician 2000). This 31‐item instrument measures the work environment across five subscales: nurse participation in hospital affairs (9 items); nursing foundations for quality care (10 items); nurse manager leadership, ability and support (5 items); adequate staffing and resources (4 items); collegial nurse‐physician relationships (3 items). Items are rated on a four‐point scale ranging from 1 (strongly disagree) to 4 (strongly agree). The mean for each of the subscales was computed to allow for comparisons with an average of 2.5 deemed a neutral midpoint (Lake 2002). A higher score was indicative of a positive work environment. The PES‐NWI has also shown good levels of reliability (Roche and Duffield 2010) and good predictive validity (Bruyneel et al. 2009). For this present study, Cronbach's alphas were: staffing and resources—Time 1 *α* = 0.52/Time 2 *α* = 0.82; collegial nurse‐physician relationships—Time 1 *α* = 0.75/Time 2 *α* = 0.78; nurse manager leadership, ability and support—Time 1 *α* = 0.77/Time 2 *α* = 0.81; nurse participation in hospital affairs—Time 1 *α* = 0.81/Time 2 *α* = 0.89; nursing foundations for quality care—Time 1 *α* = 0.61/Time 2 *α* = 0.85.

Job satisfaction was measured by a single item on a four‐point scale (very dissatisfied to very satisfied) that asked respondents: ‘How satisfied are you with your current job in this department?’ These categories were collapsed into satisfied (very satisfied/satisfied) and dissatisfied (dissatisfied/very dissatisfied).

Intention to stay was measured by asking respondents: ‘Which of the following statements most clearly reflects your feelings about your future in the hospital? Definitely will leave; probably will leave; probably will not leave and definitely will not leave.’ These response categories were collapsed into stay (probably will not leave/definitely will not leave) and leave (definitely will leave/probably will leave).

Respondents were also asked about the quality of the care they deliver through the following item: ‘How would you describe the quality of nursing care delivered to patients in your department during the last shift?’ Response options were poor; fair; good and excellent. Categories were collapsed into good/excellent quality of nursing care and poor/fair quality of nursing care.

To determine the demographic characteristics of the respondents, data on gender, highest qualification, specialist ED qualification, position (nursing grade) and experience (number of months working at as an RN) were measured.

### 4.6 Data Analysis

Data analysis was conducted using IBM SPSS Version 26 (IBM Company, Chicago, IL, USA). Data were collected at two time‐points. There was a small number of nurses who completed the questionnaire at Time 1 and Time 2 (*n* = 62) and due to this low number we decided to treat each questionnaire time‐point independently, following the approach used by Couper et al. (2022). Two respondents did not complete any of the PES‐NWI‐R or the MBI and were eliminated from the analysis. Additionally, a small number of items had missing data in the MBI dimensions and the PES‐NWI‐R subscales at Time 1 and Time 2. Missing value analyses indicated that there were no variables with 5% or more of missing data. Item mean substitution was used to replace missing data if there were no more than five responses missing for any individual item. Descriptive statistics were used to describe the respondent's demographic and professional profiles and chi‐square analysis, independent samples *t*‐test and non‐parametric tests were conducted to compare responses at Time 1 and Time 2 (see Tables 1 and 2). The Cohen's d and Phi Coefficient effect sizes were reported.

**TABLE 1:** Profile of the sample of registered nurses at Time 1 (prior to staffing adjustments) and Time 2 (following staffing adjustments).

	Time 1 (*N* = 124)		Time 2 (*N* = 129)		*p*
	(2018)		(2020)		
	*n* (%)	Median (IQR)	*n* (%)	Median (IQR)	
Gender^a^					0.61
Female	103 (83.7)		103 (80.5)		
Male	20 (16.3)		25 (19.5)		
Qualification					0.99
Certificate	7 (5.7)		6 (4.7)		
Diploma	9 (7.4)		10 (7.9)		
Degree	49 (40.2)		52 (40.9)		
PG Certificate	8 (6.6)		9 (7.1)		
PG Diploma	40 (32.7)		41 (32.3)		
Masters	9 (7.4)		9 (7.1)		
Specialist ED Qualification^a^	55 (45.8)		62 (48.4)		0.78
Nursing Grade					0.62
Staff Nurse	93 (75.6)		93 (72.1)		
Nurse Manager	30 (24.4)		36 (27.9)		
Number of months working as a Registered Nurse^b^		110.00 (65.25, 208.00)		112.50 (75.00, 186.00)	0.80
0–12 months (inclusive) nursing experience^a^	6 (4.8)		3 (2.3)		0.46

^a^ Yates continuity correction reported.

^b^ Number of months working as a registered nurse—non‐parametric test.

**TABLE 2:** Burnout, work environment, job satisfaction, intention to stay and quality of care delivered prior to (Time 1) and following (Time 2) staffing adjustments.

	Time 1 (2018)			Time 2 (2020)			*p*	Mean difference	95% CI of the difference	Effect size (95% CI)
	Mean	SD	*n* (%)	Mean	SD	*n* (%)				
MBI^a^										
Emotional exhaustion	30.73	11.47		25.60	10.93			**5.13**	2.35, 7.92	0.46 (0.21, 0.71)^c^
Depersonalization	11.59	6.82		10.12	6.34			1.47	−0.16, 3.11	0.22 (−0.02, 0.47)^c^
Personal accomplishment	33.98	8.11		33.16	7.82			0.82	−1.16, 2.80	0.10 (−0.14, 0.35)^c^
PES—NWI^b^										
Staffing and resource adequacy	1.78	0.76		2.17	0.63			**−0.39**	−0.56, −0.21	−0.55 (−0.81, −0.30)^c^
Collegial nurse doctor relations	2.94	0.58		3.13	0.52			**−0.19**	−0.32, −0.05	−0.61 (−0.87, −0.35)^c^
Nurse manager ability, leadership and support	2.65	0.54		2.85	0.57			**−0.20**	−0.34, −0.06	−0.36 (−0.61, −0.12)^c^
Nurse participation in hospital affairs	2.44	0.47		2.66	0.56			**−0.22**	−0.35, −0.09	−0.43 (−0.68, −0.18)^c^
Nursing foundations for quality of care	2.59	0.54		2.73	0.47			**−0.15**	−0.27, −0.02	−0.29 (−0.54, −0.04)^c^
Job Satisfaction—Satisfied			63 (51.6)			101 (78.3)	**< 0.01**			0.28^d^
Intention to Stay			59 (48.4)			68 (52.7)	0.57			0.04^d^
Quality of Care Delivered							**< 0.01**			0.23^d^
Poor			12 (10.0)			2 (1.6)				
Fair			48 (40.0)			42 (32.5)				
Good			53 (44.2)			68 (52.7)				
Excellent			7 (5.8)			17 (13.2)				

*Note:* Values in bold were statistically significance at the *p* < 0.05 level.

^a^ Theoretical Range for MBI—Emotional Exhaustion 0–54; Depersonalization 0–30; Personal Accomplishment 0–48.

^b^ Theoretical Range for PES‐NWI‐R subscales 0–4.

^c^ Cohen's *d*.

^d^ Phi.

### 4.7 Ethical Considerations

Ethical approval for the study was granted by two different ethics committees representing the three research sites: the Clinical Research Ethics Committee of the Cork Teaching Hospitals (Reference number ECM4 (u) Date 14/08/2018) and University Hospital Institutional Review Board (Reference number 1/378/2009 Date 12/10/2018). The researchers delivered information seminars to the ED nurses at each of the sites where the detail of the research was explained, and queries addressed. The return of completed questionnaires indicated consent to participate in the study.

## 5 Results

### 5.1 Characteristics of the Sample

There was a total of 253 responses (Time 1–124 and Time 2–129) with no significant difference between gender, qualifications, position or experience between the two time‐points (Table 1). The majority of responses at both time‐points were from nurses who identified as female (83.7% at Time 1% and 80.5% at Time 2) with over 40% of the sample at both Time 1 and Time 2 having degree level or higher qualifications. Just under half of the respondents reported that they held a specialist ED nursing qualification at both time‐points.

Table 2 shows respondents' mean scores for burnout (emotional exhaustion, depersonalisation, personal accomplishment), the five subscales on the PES‐NWI‐R and the proportion of respondents who were satisfied in their job, intended to stay and ratings of quality of care delivered in their ED at time‐points prior to (Time 1) and following (Time 2) the adjustments to nurse staffing.

Emotional exhaustion scores were lower (better) with a mean difference of 5.13 (95% CI of the difference 2.35, 7.92) following the implementation of the staffing adjustments reducing from a mean of 30.73 (SD = 11.47) at Time 1 to a mean of 25.60 (SD = 10.93) at Time 2 (theoretical range 0–54). The 95% CI for the mean difference is centred around the mean reflecting accurate representation and indicates a significant difference in emotional exhaustion between Time 1 and Time 2. There was a small to medium effect size (Cohen's d) of 0.46 (95% CI [0.21, 0.71]).

Mean scores for depersonalization were 11.59 (SD = 6.82) at Time 1 and 10.12 (SD = 6.34) at Time 2 (theoretical range 0–30). The mean difference of 1.47 (95% CI of the difference − 0.16, 3.11) was not significantly different. The Cohen's d effect size was small at 0.22 (95% CI [−0.02, 0.47]). Similarly, although there was a reduction in the mean scores for personal accomplishment (theoretical range 0–48) from Time 1 (mean 33.98, SD = 8.11) to Time 2 (mean 33.16, SD = 7.82), with a mean difference of 0.82 (95% CI of the difference − 1.16, 2.80) this was not statistically significant. There was a negligible and insignificant Cohen's d of 0.10 (95% CI [−0.14, 0.35]).

All subscales of the PES‐NWI‐R (theoretical range 0–4) had significantly higher mean scores (better) in Time 2 (following the staffing adjustments) compared with Time 1 with mean differences ranging from −0.15 to −0.39. There was an increase in the respondents' perceptions of the quality of collegial nurse doctor relations from Time 1 to Time 2 (mean difference − 0.19, 95% CI of the difference − 0.32, −0.05) with a medium effect size at −0.61 (95% CI [−0.87, −0.35]). In addition, staff perceptions of staffing and resource adequacy mean scores also improved following the staffing adjustments (Time 1 Mean = 1.78 (SD = 0.76); Time 2 Mean = 2.17 (SD = 0.63)), with a mean difference of −0.39 (95% CI of the difference − 0.56, −0.21) and a medium effect size of −0.55 (95% CI [−0.81, −0.30]). The three remaining subscales also improved following the adjustments but not to the extent of the previous subscales of the PES‐NWI‐R: nurse participation in hospital affairs had a small to medium effect size of −0.43 (95% CI [−0.68, −0.18]) followed by nurse manager ability, leadership and support with a Cohen's d of −0.36 (95% CI [−0.61, −0.12]) and nurse foundations for quality of care (Cohen's d of −0.29, 95% CI [−0.54, −0.04]).

Respondents' reports of job satisfaction increased substantially following the introduction of the staffing adjustments to meet patient need, increasing from 51.6% stating that they were satisfied or very satisfied in their job at Time 1 compared to 78.3% at Time 2. Phi Coefficient of 0.28 shows a small to medium association between time and job satisfaction. The quality of care delivered was also perceived to improve following the implementation of the adjustments to nurse staffing, increasing from 50% of respondents rating care as good or excellent at Time 1 to 65.9% at Time 2. Phi Coefficient of 0.23 indicated a small to medium association between time and quality of care delivered. Although there was a slight increase in the proportion of staff who indicated that they intended to stay over the two time‐points (Time 1 = 48.4%; Time 2 = 52.7%), this change was not statistically significant with no or a very small association identified between time and intention to stay.

## 6 Discussion

Implementing a planned change to nurse staffing levels based on patient need in three EDs resulted in reduced levels of emotional exhaustion (burnout), improved perceptions of the work environment, increased job satisfaction and perceived improvement in the delivery of quality care in a sample of ED nurses. This study is one of the first to measure the impact of planned changes to staffing, based on patient need, on nursing staff working in ED. The rationale for introducing the changes was based on the growing demands on nurses in EDs including increased crowding, complexity of patient presentations and issues with the recruitment and retention of nursing staff in the sector.

The improvements in staff outcomes that are shown in this research occurred after the implementation of the Framework for Safe Nurse Staffing and Skill Mix in Emergency Settings in Ireland, which was an urgent policy reform recognising the experiences of staff and patients in the ED setting (Department of Health 2022). The Framework resulted in an organisational change where nurses were, through safe staffing levels, able to deliver quality care within a better work environment. There has been previous evidence of the benefit of such work conditions in relation to the retention of nurses and delivery of quality care (Buchan and Catton 2023). However, the implementation of this policy needs to be made cognisant of other challenges in the Irish healthcare system such as long waiting lists, and inefficient use of acute hospital beds (Burke et al. 2023). Nevertheless, this study presents clear benefits of this policy reform in relation to the experiences of nurses working in ED. Implementing the Framework that supports safe staffing, adequate skill mix and clinical manager supervisory support in the ED allows the organisation to promote their workplace as an attractive area to work (Buchan and Catton 2023).

The Manchester Triage System (Mackway‐Jones et al. 2006) was used as the measure of the urgency of care for patients attending all three EDs and the number of nursing hours required for patients in each of the categories. Although the number of patient presentations over the two time‐points remained relatively unchanged (Drennan et al. 2022), the introduction of staffing levels based on patient need as determined by their triage score, reduced overall nursing workload. Reduced workloads are associated with lower levels of nursing staff burnout (Dall'Ora et al. 2020; Tsolakidis and Vasiliki Diamantidou 2022) and, as reported in this study, increases to staffing reduced levels of emotional exhaustion between the two time‐points. It is of note that, although levels of burnout reduced following the amendments to nurse staffing, burnout scores, particularly emotional exhaustion were higher when compared to normative values and previous studies (Lee et al. 2021). This demonstrates the continued stressors for nursing staff working in EDs, including the perennial issue of crowding, and concur with previous findings in a meta‐analysis for emergency nurses' experience of burnout (Gómez‐Urquiza et al. 2017). It may also be reflective of Time 2 data being collected around the time of the first positive COVID‐19 case in Ireland; however data were collected prior to an increase in patients with COVID‐19 attending the three EDs. Nevertheless, it is envisioned that the embedding of the recommendations from the Framework will facilitate a further reduction in the emotional exhaustion levels experienced by ED nurses.

Safe staffing in the ED has typically been difficult to measure. The approach to determining staffing levels (NHpPP) outlined in this paper was sensitive to workload fluctuations which allowed for a more dynamic assessment of staffing adequacy compared to static nurse to patient ratios. Our study's use of systematically collected staffing data facilitated improved measurement precision. In addition, by measuring change from Time 1 to Time 2, we were able to capture the impact of staffing modifications and the implementation of the Framework over time, providing an assessment of their effects on respondents' perceptions of the practice environment.

Research has shown that lower staffing in the ED can result in negative patient outcomes with, for example, an increase in patients leaving without being seen (Ramsey et al. 2018). This in turn can impact the ED nurse's perception of their work environment with aspects of this environment such as staffing and resource adequacy and collegial nurse physician relations being seen as priority areas to be addressed (Gasparino et al. 2021). In this paper, we did not examine patient outcomes, but the systematic approach taken to staff the EDs based on patient acuity resulted in a statistically significant improvement from Time 1 to Time 2 for emotional exhaustion suggesting a difference that could be of practical significance. Similarly, we found a statistically significant difference before and after the introduction of the Framework for subscales of the PES‐NWI‐R which indicated improvements in staffing and resource adequacy and enhanced collegiality between nurses and doctors which could result in higher levels of job satisfaction and staff retention (Alenazy et al. 2021) and better patient outcomes. Researchers held information sessions at each of the EDs to inform staff about the purpose of the study and of the potential benefits the Framework could have for their work environment (staffing adjustments, appropriate skill mix and supervisor support). There was positive engagement from staff with this research which ultimately facilitated an organisational change resulting in staffing levels required to deliver safe patient care. Organisational changes that facilitate more time delivering direct patient care have been found to result in a more satisfied healthcare workforce (Aiken et al. 2024).

Time 2 data were collected at the beginning of the COVID‐19 pandemic in Ireland; however, at the time of data collection, the EDs had not yet been impacted by admission of patients with COVID‐19. Fear of the unknown in relation to the virus, infection controls procedures, and country lock downs would have heightened stressors for ED nurses who were, very often, the first point of contact for patients entering the acute hospital setting. However, we saw an improvement in emotional exhaustion levels, work environment and job satisfaction suggesting that, despite unprecedented times, nurses reported better outcomes following adjustments to staffing based on the acuity of the patient presentation. Strategies were implemented at speed to prepare and manage healthcare delivery during COVID‐19, and this demonstrated clear leadership. This, in addition to collegial working and the provision of resources to ensure nurses could deliver care safely may have resulted in the improved emotional exhaustion levels and job satisfaction (Gasparino et al. 2021).

Attaining and maintaining the required nurse staffing based on patient acuity can be dependent on national and international factors. There is a recruitment and retention crisis for nurses worldwide. EDs, which were typically areas where a nurse needed experience to work in, are now accepting new graduates (García‐Martín et al. 2021). The planned change to nurse staffing that was made in the three EDs in this study resulted in an improvement to the perceived work environment, job satisfaction and quality of care delivered and saw a reduction between Time 1 and Time 2 in emotional exhaustion. These positive findings may help support the retention of nurses and attract new graduates to ED nursing (Buchan and Catton 2023). The importance of interventions to improve the working environment for the retention of nurses has been recognised (Aiken et al. 2024) but additionally offering opportunities in further education (specialisation in emergency nursing), and career progression will further improve retention. These initiatives will also attract new graduates to work in the ED where they will see a clear career progression pathway within a supportive work environment.

### 6.1 Limitations

ED patient numbers attending and waiting on trolleys were high at the time of the research. This is a recognised problem in Irish EDs (Burke et al. 2023). Additionally, it is possible that symptoms of burnout led to a bias in the reporting of work environment, job satisfaction and intention to stay as burnout typically ensues from unmanaged chronic workplace stress (World Health Organization 2019).

The healthy worker effect, where only nurses who were at work were surveyed, could be a limitation to this study. The design of the research excluded nurses on leave owing to them not having the opportunity to complete the questionnaire, which was available in the EDs only. This potentially resulted in lower levels of burnout being reported. Completing the questionnaire after a busy shift may also have negatively influenced responses. Nevertheless, robust, valid instruments were used to measure burnout and work environment for ED nurses across three hospitals. These performed well in general with Time 2 reliability in line with previous studies (Roche and Duffield 2010). In future research we will look at job satisfaction and intention to stay with a larger sample without collapsing the response categories. This may allow us to interpret findings more precisely in relation to these outcomes.

In some of the EDs, the full staffing adjustment was only in place for 2–3months before Time 2 data were collected. Although we did find an improvement to emotional exhaustion, and nurses' perceptions of their work environment, a longer timeline between the staffing adjustment and Time 2 data collection would have strengthened our findings. We are currently planning a follow‐up study with the three EDs so we can look at these associations over a longer period of time. The study was conducted in three different EDs which adds to the generalisability of the findings to other ED settings in Ireland, but the findings may not be generalisable to EDs globally.

### 6.2 Recommendations for Further Research

Nurse shortages impact on the recruitment and retention of ED nurses. Evidence of better experiences for ED nurses after the Framework introduction suggest that this important organisational change is making a real difference to the nurse's work experience. However, follow‐up work on the ED environment is required when there is stability of staffing to determine if this uplift will enhance and improve the experience for the ED nurse in the long term. The collection of national longitudinal data as part of the Safe Nurse Staffing and Skill Mix Framework will provide valuable evidence to inform ED staffing with anticipated and sustained improvements seen in nurse job satisfaction, burnout levels, retention and quality of care.

### 6.3 Implications for Policy and Practice

There was an uplift of 27.91 FTEs RNs across the three EDs in this study. We have shown an improvement to nurse outcomes demonstrating that the Framework is an important policy reform with clear advantages in practice. However, an upward adjustment of ED staff has financial implications and needs to be reviewed regularly. With this in mind, data on the number of patients presenting in the ED in addition to their triage category should be routinely collected to reassess staffing levels every 6 months (Department of Health 2022). This reassessment will account for seasonal variation and highlight any under or over staffing. The Framework for Safe Nurse Staffing and Skill Mix in Adult Emergency Care Settings in Ireland was brought into policy in the Republic of Ireland in recognition of the acuity of the patient presenting to ED and the nursing care required. It is an important organisational change that results in staffing based on patient acuity allowing for safer, higher quality care to be delivered. The cost associated with the Framework introduction needs to be weighed against the cost of replacing staff (Bae 2022) who may leave due to poor work environments. Safe staffing will allow greater job satisfaction and commitment from the nurse to their organisation.

## 7 Conclusion

The planned change to nurse staffing levels in three EDs resulted in reports of a better work environment and ED nurses more satisfied with their job. It is important, however, to allow time for changes to be implemented over a longer time‐period to influence practice in work environments. Follow up research with this cohort will be insightful in relation to the impact of staffing EDs based on patient acuity on patient, nurse and organisational outcomes. This could inform a more holistic organisational change that is required to address the work environment and burnout levels of nurses in ED. Initiatives such as involving staff in decisions around their work environment, development of their practice area to ensure it is a workable space, fostering a culture of quality improvement that is front line staff driven, will promote a pride in work and improve the experiences of the ED nurse.

## Ethics Statement

Ethical approval for the study was granted by the research ethics committees representing the three research sites (ECM4 (u) 14/08/18; 1/378/2009).

## Conflicts of Interest

The authors declare no conflicts of interest.

## Data Availability

Relevant fieldwork permission was obtained to conduct this research. All data utilised in this submitted manuscript has been lawfully acquired.

## References

[jan16845-bib-0001] Aiken, L. H. , and P. A. Patrician . 2000. “Measuring Organizational Traits of Hospitals: The Revised Nursing Work Index.” Nursing Research 49, no. 3: 146–153. 10882319 10.1097/00006199-200005000-00006

[jan16845-bib-0002] Aiken, L. H. , W. Sermeus , M. McKee , et al. 2024. “Physician and Nurse Well‐Being, Patient Safety and Recommendations for Interventions: Cross‐Sectional Survey in Hospitals in Six European Countries.” BMJ Open 14: e079931. 10.1136/bmjopen-2023-079931. PMC10862305 38346890

[jan16845-bib-0003] Al‐Dweik, G. , and M. Ahmad . 2020. “The Effectiveness of Patient Acuity Tool on Nursing and Patients Outcomes: A Literature Review.” International Medical Journal 25, no. 1: 13412051.

[jan16845-bib-0004] Alenazy, F. S. , Z. Dettrick , and S. Keogh . 2021. “The Relationship Between Practice Environment, Job Satisfaction and Intention to Leave in Critical Care Nurses.” Nursing in Critical Care 28, no. 2: 167–176. 10.1111/nicc.12737. 34882918

[jan16845-bib-0005] Bae, S.‐H. 2022. “Noneconomic and Economic Impacts of Nurse Turnover in Hospitals: A Systematic Review.” International Nursing Review 69, no. 3: 392–404. 10.1111/inr.12769. 35654041 PMC9545246

[jan16845-bib-0006] Bruyneel, L. , K. van den Heede , L. Diya , L. Aiken , and W. Sermeus . 2009. “Predictive Validity of the International Hospital Outcomes Study Questionnaire: An RN4CAST Pilot Study.” Journal of Nursing Scholarship 41, no. 2: 202–210. 10.1111/j.1547-5069.2009.01272.x. 19538705 PMC2739088

[jan16845-bib-0007] Buchan, J. , and H. Catton . 2023. “Recover to Rebuild. Investing in the Nursing Workforce for Health System Effectiveness.” ICN_Recover‐to‐Rebuild_report_EN.pdf.

[jan16845-bib-0008] Burke, C. , E. Dolan , J. Faul , et al. 2023. “Delayed Hospital Discharges and the Trolley Crisis.” Irish Journal of Medical Science 192: 11–14. 10.1007/s11845-022-02924-z. 35182289

[jan16845-bib-0009] Butler, M. , T. J. Schultz , P. Halligan , et al. 2019. “Hospital Nurse‐Staffing Models and Patient‐and Staff‐Related Outcomes (Review).” Cochrane Database of Systematic Reviews 4: CD007019. 10.1002/14651858.CD007019.pub3. 31012954 PMC6478038

[jan16845-bib-0010] Caulfield, R. , T. Wiseman , J. Gullick , and R. Ogilvie . 2023. “Factors preceding occupational distress in emergency nurses: An integrative review.” Journal of Clinical Nursing 32, no. (13‐14): 3341–3360. 35871282 10.1111/jocn.16461

[jan16845-bib-0011] Couper, K. , T. Murrells , J. Sanders , et al. 2022. “The Impact of COVID‐19 on the Wellbeing of the UK Nursing and Midwifery Workforce During the First Pandemic Wave: A Longitudinal Survey Study.” International Journal of Nursing Studies 127: 104155. 10.1016/j.ijnurstu.2021.104155. 35093740 PMC8673915

[jan16845-bib-0012] Dall'Ora, C. , J. Ball , M. Reinius , and P. Griffiths . 2020. “Burnout in Nursing: A Theoretical Review.” Human Resources for Health 18, no. 1: 1–17. 10.1186/s12960-020-00469-9. 32503559 PMC7273381

[jan16845-bib-0013] de Wijn, A. N. , M. Fokkema , and M. P. van der Doef . 2022. “The Prevalence of Stress‐Related Outcomes and Occupational Well‐Being Among Emergency Nurses in The Netherlands and the Role of Job Factors: A Regression Tree Analysis.” Journal of Nursing Management 30, no. 1: 187–197. 10.1111/jonm.13457. 34448288 PMC9290041

[jan16845-bib-0014] Department of Health . 2022. “Framework for Safe Nurse Staffing and Skill Mix in Adult Emergency Care Settings in Ireland. Final Report and Recommendations by the Taskforce on Staffing and Skill Mix for Nursing.” http://www.gov.ie/pdf/?file=https://assets.gov.ie/226687/1a13b01a‐83a3‐4c06‐875f‐010189be1e22.pdf#page=null.

[jan16845-bib-0015] Dillman, D. 1978. Mail and Telephone Surveys: The Total Design Method. John Wiley & Sons.

[jan16845-bib-0016] Drennan, J. , A. Murphy , V. J. C. Mc Carthy , et al. 2024. “The Association Between Nurse Staffing and Quality of Care in Emergency Departments: A Systematic Review.” International Journal of Nursing Studies 153: 104706. 10.1016/j.ijnurstu.2024.104706. 38447488

[jan16845-bib-0017] Drennan, J. , E. Savage , J. Hegarty , et al. 2022. “Evaluation of the Pilot Implementation of the Framework for Emergency Care Settings—Report 2.” https://www.gov.ie/pdf/?file=https://assets.gov.ie/135001/538eaf16‐e721‐458f‐8cb2‐46b5c5afea7c.pdf#page=null.

[jan16845-bib-0018] García‐Martín, M. , P. Roman , M. Rodriguez‐Arrastia , M. d. M. Diaz‐Cortes , P. J. Soriano‐Martin , and C. Ropero‐Padilla . 2021. “Novice Nurse's Transitioning to Emergency Nurse During COVID‐19 Pandemic: A Qualitative Study.” Journal of Nursing Management 29, no. 2: 258–267. 10.1111/jonm.13148. 32881134

[jan16845-bib-0019] Gasparino, C. R. , D. M. Ferreira , H. Ceretta Oliveira , D. Fernanda Dos Santos Alves , and A. Pazetto Balsanelli . 2021. “Leadership, Adequate Staffing and Material Resources, and Collegial Nurse–Physician Relationships Promote Better Patients, Professionals and Institutions Outcomes.” Journal of Advanced Nursing 77, no. 6: 2739–2747. 10.1111/jan.14805. 33624302

[jan16845-bib-0020] Gómez‐Urquiza, J. L. , E. I. De la Fuente‐Solana , L. Albendín‐García , C. Vargas‐Pecino , E. M. Ortega‐Campos , and G. A. Cañadas‐De la Fuente . 2017. “Prevalence of Burnout Syndrome in Emergency Nurses: A Meta‐Analysis.” Critical Care Nurse 37, no. 5: e1–e9. 10.4037/ccn2017508. 28966203

[jan16845-bib-0021] Lake, E. T. 2002. “Development of the Practice Environment Scale of the Nursing Work Index.” Research in Nursing & Health 25, no. 3: 176–188. 10.1002/nur.10032. 12015780

[jan16845-bib-0022] Lee, M. M. D. , M. M. Gensimore , R. S. Maduro , M. K. Morgan , and K. S. Zimbro . 2021. “The Impact of Burnout on Emergency Nurses' Intent to Leave: A Cross‐Sectional Survey.” Journal of Emergency Nursing 47, no. 6: 892–901. 10.1016/j.jen.2021.07.004. 34417028

[jan16845-bib-0023] Mackway‐Jones, K. , J. Marsden , and J. Windle , eds. 2006. Emergency Triage Manchester Triage Group. 2nd ed. Blackwell Publishing Ltd.

[jan16845-bib-0024] Maslach, C. , and S. E. Jackson . 1996. Maslach Burnout Inventory–Human Services Survey (MBI–HSS). 3rd ed. Consulting Psychologist Press.

[jan16845-bib-0025] McDermid, F. , J. Mannix , and K. Peters . 2020. “Factors Contributing to High Turnover Rates of Emergency Nurses: A Review of the Literature.” Australian Critical Care 33, no. 4: 390–396. 10.1016/j.aucc.2019.09.002. 31836449

[jan16845-bib-0026] Morley, C. , M. Unwin , G. M. Peterson , J. Stankovich , and L. Kinsman . 2018. “Emergency Department Crowding: A Systematic Review of Causes, Consequences and Solutions.” PLoS One 13, no. 8: e0203316. 10.1371/journal.pone.0203316. 30161242 PMC6117060

[jan16845-bib-0027] Norful, A. A. , K. Cato , B. P. Chang , T. Amberson , and J. Castner . 2023. “Emergency Nursing Workforce, Burnout, and Job Turnover in the United States: A National Sample Survey Analysis.” Journal of Emergency Nursing 49, no. 4: 574–585. 10.1016/j.jen.2022.12.014. 36754732 PMC10329980

[jan16845-bib-0028] Ramsey, Z. , J. S. Palter , J. Hardwick , J. Moskoff , E. L. Christian , and J. Bailitz . 2018. “Decreased Nursing Staffing Adversely Affects Emergency Department Throughput Metrics.” Western Journal of Emergency Medicine 19, no. 3: 496–500. 10.5811/westjem.2018.1.36327. 29760847 PMC5942016

[jan16845-bib-0029] Roche, M. A. , and C. M. Duffield . 2010. “A Comparison of the Nursing Practice Environment in Mental Health and Medical‐Surgical Settings.” Journal of Nursing Scholarship 42, no. 2: 195–206. 10.1111/j.1547-5069.2010.01348.x. 20618603

[jan16845-bib-0030] Tsolakidis, G. , and M. D. Vasiliki Diamantidou . 2022. “Nursing Staff Burnout: A Critical Review of the Risk Factors.” International Journal of Caring Sciences 15, no. 1: 668–679.

[jan16845-bib-0031] Wise, S. , M. Fry , C. Duffield , M. Roche , and J. Buchanan . 2015. “Ratios and Nurse Staffing: The Vexed Case of Emergency Departments.” Australasian Emergency Nursing Journal 18, no. 1: 49–55. 10.1016/j.aenj.2014.08.001. 25241011

[jan16845-bib-0032] World Health Organization . 2019. “Burn‐Out an “Occupational Phenomenon”: International Classification of Diseases.” Bulletin of the World Health Organization 97, no. 9: 585–586.31474769

[jan16845-bib-0033] Youd, J. 2015. “Workforce Planning for Urgent Care Services.” Emergency Nurse 23, no. 4: 1477. 10.7748/en.23.4.14.e1477. 26159344

